# Mr. Abernethy

**Published:** 1831

**Authors:** 


### LXXVI.

### MR. ABERNETHY.

SINCE the last number of this Journal was published, that strange compound of talent and eccentricity, of enlightened observation and hobby-horsical empiricism, of goodness of heart and rudeness of manner, of amiable feeling and irritable temper, has paid the debt of Nature. It is greatly to be doubted whether his eccentricity and roughness of manner were not assumed at first, for some whim or humour, and continued afterwards, from inclination—habit —or perhaps DESIGN. This last expression will sound strange in the ears of those who believed, or rather were convinced that the late Mr. Abernethy lost many thousands annually by his MANNER of treating patients. We never participated in this opinion, nor do we believe that the individual himself did so. We firmly believe that this same rudeness drew more visitors from curiosity than it deterred by fear of insult. Let it be remembered that it caused the man to be talked of everywhere—and this very circumstance, leaving all ability out of the question, was sufficient to make his fortune. This may be illustrated by one or two very familiar examples. Dr. Eady merely writes his *name* on the walls—and yet we may be assured that he finds his account in it, or he would not take even that trouble. So with Warren's "BLACKING," and Hunt's "MATCHLESS COMPOSITION." The very sight of a name or a thing generates, by constant iteration, a species of fame or celebrity. But with a man like Mr. Abernethy, who was attached to a great hospital, and who had really distinguished himself by theoretical and practical researches, publicity was every thing—and especially when that publicity was connected with eccentricity. Whatever an eccentric character says or does is considered clever, although the same sayings or doings would pass for nothing, or for dulness, if said or done by others. In respect to medical men, there is a universal propensity in the public mind to exaggerate the benefits received from them, whenever their names happen to become familiar with the public ear. The failures, nay, even the blunders, of such men are not merely soon forgotten, but they are seldom believed at all. The routinism to which Mr. Abernethy gave way, for many years before his death, was generally ludicrous, but sometimes tragical. We have seen more than one instance where life was, in all human probability, sacrificed by an obstinate disregard of all examination of the case, and a blind perseverance in one system of treatment totally inapplicable to the existing disease. We believe, however, that when

Mr. A. could be brought to think and reason on a case, (which was a rare occurrence of late years,) his judgment was clear and his practice judicious. The eccentric sayings and doings of this talented individual would fill a volume, and some piquant specimens have been collected together in the second number of the Metropolitan Magazine for June last.

Though highly irritable in his temper, we believe Mr. A. was amiable, good-natured, and possessed of strong feelings. He was an honest as well as able surgeon, and in his eccentricities he never injured or attempted to injure, the character or interest of his professional neighbour.

Requiescat in pace!

